# Proteomic insights into survival strategies of *Escherichia coli* in perchlorate-rich Martian brines

**DOI:** 10.1038/s41598-025-91562-3

**Published:** 2025-02-27

**Authors:** Lea D. F. Kloss, Joerg Doellinger, Anne Gries, Elisa Soler, Peter Lasch, Jacob Heinz

**Affiliations:** 1 Center for Astronomy and Astrophysics, RG Astrobiology, Technische Universität Berlin, Berlin, Germany; 2 Centre for Biological Threats and Special Pathogens, Proteomics and Spectroscopy (ZBS6), Robert Koch-Institute, Berlin, Germany; 3 Present Address: Institute for Computer Science and Department of Biology, Heinrich Heine University, Düsseldorf, Germany

**Keywords:** Mars, Perchlorate, Brines, Habitability, Adaptation, Proteomics, Astrobiology, Bacteriology

## Abstract

Brines, potentially formed by the deliquescence and freezing point depression of highly hygroscopic salts, such as perchlorates (ClO_4_^−^), may allow for the spatial and temporal stability of liquid water on present-day Mars. It is therefore of great interest to explore the microbial habitability of Martian brines, for which our current understanding is, however, still limited. Putative microbes growing in the perchlorate-rich Martian regolith may be harmed due to the induction of various stressors including osmotic, chaotropic, and oxidative stress. We adapted the model organism *Escherichia coli* to increasing sodium perchlorate concentrations and used a proteomic approach to characterize the adaptive phenotype. Separately, the microbe was adapted to elevated concentrations of sodium chloride and glycerol, which enabled us to distinguish perchlorate-specific adaptation mechanisms from those in response to osmotic, ion and water activity stress. We found that the perchlorate-specific stress response focused on pathways alleviating damage to nucleic acids, presumably caused by increased chaotropic and/or oxidative stress. The significant enrichments that have been found include DNA repair, RNA methylation and de novo inosine monophosphate (IMP) biosynthesis. Our study provides insights into the adaptive mechanisms necessary for microorganisms to survive under perchlorate stress, with implications for understanding the habitability of Martian brines.

**Supplementary Information:**

The online version contains supplementary material available at 10.1038/s41598-025-91562-3.

## Introduction

While Mars likely had an abundant supply of water billions of years ago^1^, the planet’s magnetic field has since been lost, which caused atmospheric erosion^2^ and ultimately led to the instability of liquid water on its surface^3^. As a result, Mars is now known for its dry and inhospitable environment, with water existing in the form of ice^4,5^ and vapor^6^. Nevertheless, one theory of how liquid water may be present on Mars today is deliquescence^7^, a process describing how hygroscopic salts dissolve in water absorbed from the atmosphere. These salts also cause intense freezing point depressions^8^, resulting in the formation of liquid brines at subzero temperatures. The potential for the temporal and local stability of liquid brines on Mars was demonstrated experimentally under simulated Martian conditions^8,9^. Direct observational evidence on Mars includes seasonally recurring slope lineae (RSL)^10,11^ and spheroids on a strut of the Phoenix leg^12^, although alternative underlying processes have been proposed^12,13^. Furthermore, various hygroscopic salts have been detected in the Martian soil^14^, with perchlorates (ClO_4_^−^) being a prominent example^15^.

An intriguing question is therefore how habitable these liquid brines are for microbial life. Firstly, because they represent one of the last resorts of Martian life that may have evolved billions of years ago and which would have had to adapt to the desiccation of the planet^16^. And additionally, because of putative future human missions to Mars, for which perchlorate-resistant strains as well as bioremediation processes might be of interest for in-situ resource utilization (ISRU)^17,18^. However, biological systems growing in the Martian regolith may be harmed by high concentrations of perchlorates. As for any salt, these can lead to hyperosmotic stress as well as reduced water activity, which is a measure for the amount of water molecules available to microorganisms in a given system^19^. Additionally, perchlorates may have ion-specific effects on an organism’s metabolism^20,21^ and are highly chaotropic^22^. Chaotropic solutes interact in complex ways with biomacromolecules in aqueous solutions^23^. Consequently, they can disorder, destabilize and denature biochemically important structures such as proteins, nucleic acids and lipid bilayers. They can also trigger an associated secondary oxidative stress response^24,25^. This is of importance, as the role of oxidative stress in perchlorate toxicity is not yet fully understood^26^. The perchlorate anion has a tetrahedral structure with a chlorine atom of oxidation state +7 at its center. Although it is therefore thermodynamically considered a strong oxidant, it actually has a low reactivity in ambient conditions, due to its reduction being kinetically inhibited^27^.

While widespread on Mars, perchlorates are rarely found naturally on Earth, which is why life in perchlorate-rich environments remains to be elucidated in more detail. It was detected in several arid regions^28^ at mostly lower concentrations than on Mars^29^, which have been reported to be 0.4–0.6 wt%^30^. On Earth, this is only matched by the Atacama Desert in Chile, where perchlorate concentrations of up to 0.6 wt% were detected in nitrate ores^29,31^. Several organisms have been identified that are capable of the enzymatic reduction of perchlorate to chloride under anaerobic conditions, which is why they may be relevant for bioremediation purposes^32^. However, the perchlorate-tolerance as well as adaptation mechanisms to perchlorates are also of importance and its investigation has increased in recent years. The halotolerant yeast *Debaryomyces hansenii* has the highest perchlorate-tolerance reported to date with 2.5 mol/kg (23 wt%)^33^, which is especially relevant considering that perchlorates form highly concentrated brines under deliquescence^21,34^. As an adaptive strategy it stabilizes biomacromolecules via protein glycosylation and cell wall remodulation, which likely aims to counteract perchlorate-induced chaotropic stress^26^. Furthermore, a metagenomics approach was used to search for novel perchlorate-resistance mechanisms in microbes from sediments of a hypersaline pond in the Atacama Desert^35^. This has resulted in the identification of genes involved in diverse cellular processes, including nucleic acid and protein degradation. Several of these were also associated with an increased tolerance to oxidative stress.

The aforementioned studies examined a halotolerant eukaryote as well as genes from most likely halophilic microbial communities. It however remains to be elucidated how a non-halotolerant prokaryote would deal with increased perchlorate-concentrations in its environment and which adaptive strategies it would pursue. Non-halotolerant organisms are not inherently adapted to saline conditions, making their stress responses more pronounced and easier to analyze. These responses can offer clues about the initial steps and mechanisms required for adaptation to such extreme environments, providing a baseline for understanding potential evolutionary pathways for life under Mars-like conditions. This approach allows us to predict which physiological or molecular adaptations might have been relevant for ancient Martian microbes as the planet underwent prolonged desiccation and salinization. In addition, studying non-halotolerant organisms allow for direct comparisons with halotolerant organisms, highlighting unique versus shared stress responses. This may allow the identification of universal strategies for coping with high salt and perchlorate levels, as well as specific adaptation mechanisms for halotolerant and non-halotolerant organisms.

To address this research question, we used adaptive laboratory evolution of the well-established model-organism *Escherichia coli* to increasing concentrations of sodium perchlorate (NaClO_4_). We complemented this with a proteomic approach combining unbiased SPEED-based sample preparation and data-independent acquisition mass spectrometry (DIA-MS)^36,37^. Adaptive evolution provides information on long-term survival strategies and the large number of existing studies on *E. coli* as well as its high adaptation potential allow us to put the adaptive phenotype into a comprehensive context. These findings are also relevant for ISRU applications. Non-halotolerant organisms like *E. coli* may show the extent of stress adaptations needed to function in saline environments, potentially guiding the engineering of robust microbial systems for resource utilization on Mars. In addition, engineered *E. coli* have already been demonstrated to be applicable for ISRU^38^.

Since perchlorate induces different types of stress, we separately adapted *E. coli* to increasing NaCl and glycerol concentrations. The present study focuses on the phenotypic changes that occur after acclimation, which are most likely attributable to phenotypic plasticity. The role of genetic mutations has not been further evaluated and is presumably negligible. The acclimation to increasingly higher solute concentrations is here referred to as adaptation. By comparing the individual adaptive phenotypes, we were able to distinguish perchlorate-specific stress responses from those in response to osmotic, ion and water activity stress. This study advances our understanding of adaptation mechanisms necessary for life to thrive in perchlorate-rich environments, and thus for the habitability of Martian brines.

## Materials and methods

### Organism and culture conditions

*E. coli* DSM 498 (K-12 ‘wildtype’) was used for this study and was obtained from the Leibniz Institute DSMZ—German Collection of Microorganisms and Cell Cultures (Braunschweig, Germany). The cultures were grown aerobically as liquid cultures in DSMZ growth medium #1 (5 g/L peptone, 3 g/L meat extract, pH 7.0–7.5) and incubated at 35 °C without shaking.

### Adaptation to elevated solute concentrations

**Fig. 1:**
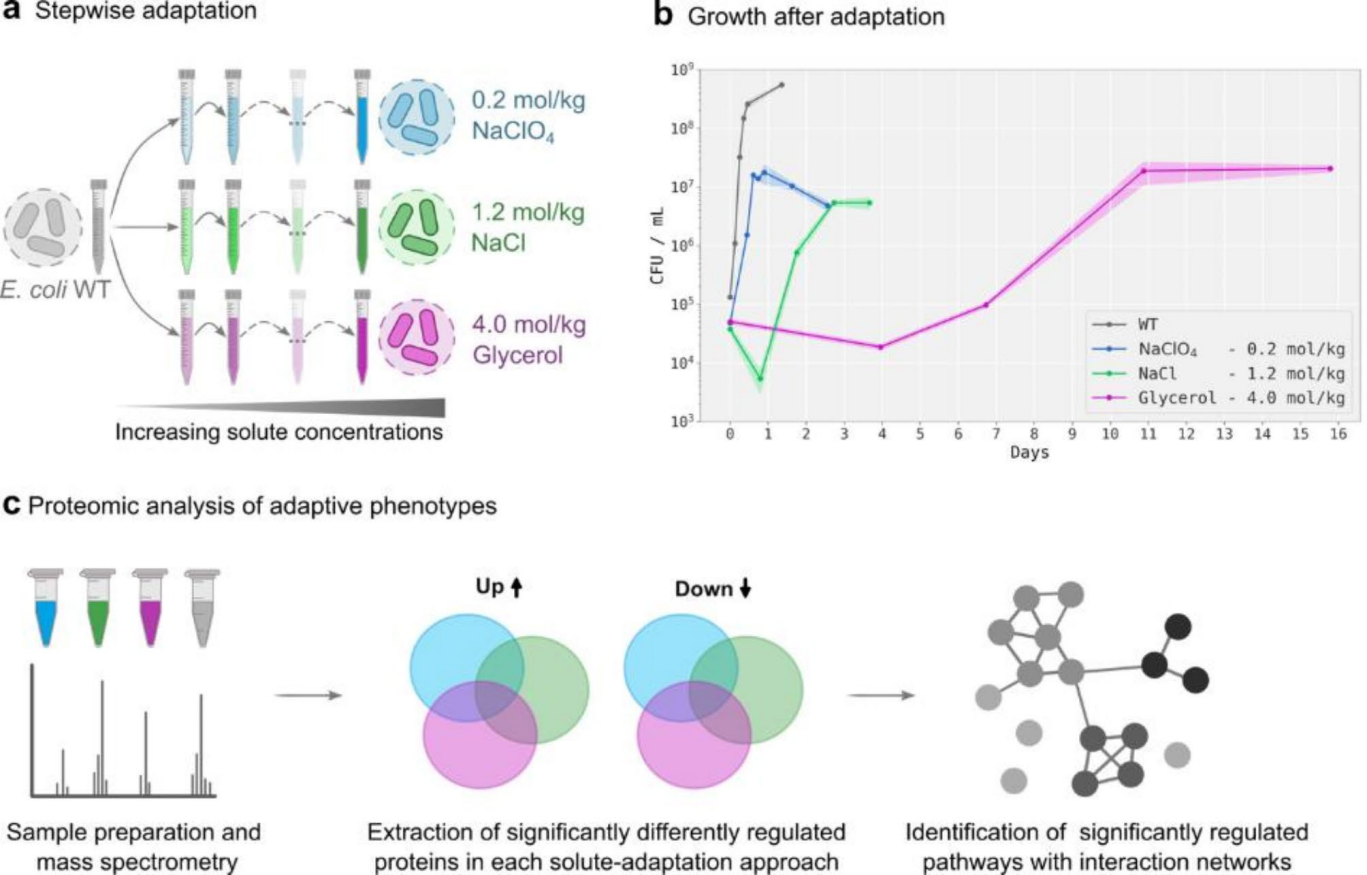
Schematic illustration of the experimental workflow. (**a**) *E. coli* was incrementally adapted to increasing concentrations of NaClO_4_, NaCl and glycerol. (**b**) Example growth profiles of wild-type (WT) *E. coli* (grey) and after its adaptation and subsequent propagation at concentrations of 0.2 mol/kg NaClO_4_ (blue), 1.2 mol/kg NaCl (green) and 4 mol/kg glycerol (pink). Data represent means ± SD (*n* = 3). (**c**) The adaptive phenotypes were analyzed using proteomics. First, triplicates of each treatment and the WT were prepared, harvested during late exponential growth and analyzed by mass spectrometry. Next, we aimed to identify those adaptation mechanisms, which were specific for perchlorate-induced stress in *E. coli*. For this, a post hoc test (FDR ≤ 0.01) was performed, giving information on which treatment combinations significantly differ from each other for each protein. Sets of proteins for solute-specific and shared adaptation mechanisms were extracted using a strict filtering process and graphically depicted as Venn diagrams (see Materials and Methods). Lastly, the protein sets were used to identify significant adaptive pathways (FDR ≤ 0.05) using protein-protein interaction networks with the help of the STRING database^42^.

*E. coli* was incrementally adapted to increasingly higher concentrations of NaClO_4_, NaCl and glycerol in order to identify the individually induced stress responses and allow the identification of perchlorate-specific stress responses (Fig. 1). To adapt *E. coli* to elevated solute concentrations, the growth medium DSMZ #1 was supplemented with increasing concentrations of NaClO_4_, NaCl or glycerol with each new inoculation step (Fig. 1a). The increments of concentration increase ranged between 0.025 and 1.0 mol/kg depending on the type of solute and the observed growth patterns. Cell growth and death were documented by measuring the optical density at 600 nm (OD_600_) and counting colony forming units (CFUs) on DSMZ #1 agar plates (5 g/L peptone, 3 g/L meat extract, 15 g/L agar, pH 7.0–7.5). Inoculation into the next higher concentration was carried out after the cells reached late exponential or stationary growth and starting cell densities were targeted to be between 10^2^ and 10^4^ CFU/mL. We never propagated the cells repeatedly in the same solute concentration during the adaptation process, except for 3.25 mol/kg glycerol, which was repeatedly cultured once, as the first attempt to increase the solute concentration had to be repeated due to insufficient cell concentration at the start. The solute concentrations were increased until a concentration was reached for which no reproducible growth could be achieved within a time frame of approximately 3 weeks. After reaching these final concentrations, the adapted cultures were transferred multiple times into fresh media with the same elevated solute concentrations. This was done to verify the reproducibility of the growth curves through repeated inoculations. The process was continued until the point in the growth curve corresponding to the late exponential phase could be reliably identified for proteome sampling. For future experiments, it would be beneficial to investigate how significantly the number of inoculation cycles at the same elevated solute concentrations influence the proteomic stress responses. Statistical growth analyses of triplicates were carried out for the concentrations chosen for proteome analysis of each solute (Fig. 1b), whereas growth curves for each concentration during adaptation were measured for single liquid cell cultures (Supplementary Fig. S1).

### Protein extraction

Triplicates of cell cultures adapted to grow at 0.2 mol/kg NaClO_4_, 1.2 mol/kg NaCl and 4 mol/kg glycerol, as well as of the WT in DSMZ #1 without supplementation of additional solutes (control), were harvested during the late exponential growth phase, as determined by OD_600_ and CFU.

The protein extraction was carried out based on the chemical Sample Preparation by Easy Extraction and Digestion (SPEED) protocol^36^. The cell cultures were centrifuged at 3000 × *g* for 15 min. The pellets were washed three times with 1 mL PBS, vortexed and centrifuged at 3000 × *g* for 5 min. The pellets were then resuspended in 50 µL trifluoroacetic acid (TFA) for cell lysis and protein extraction. After incubation for 2–5 min at room temperature, the cell lysates were neutralized with 500 µL 2 M tris(hydroxymethyl)aminomethane (TRIS). 50 µL alkylation and reduction buffer (28.57 wt% tris(2-carboxyethyl)phosphine and 37.04 wt% 2-chloroacetamide) were added to the lysates, after which they were incubated at 95 °C for 3 min. The buffer caused disulfide bond breakage by reduction and prevention of disulfide bond reformation by alkylation. Protein concentrations were then determined by measurements of the optical density at 360 nm (NanoPhotometer NP80, Implen). Proteins were precipitated by diluting 20–90 µg protein with acetone to a final concentration of 80% acetone. One washing step was performed with 80% acetone, after which 2 µg Trypsin (Trypsin Gold, Mass Spectrometry Grade, Promega) were added. For this, 400 µL of a 5 µg/mL trypsin solution in digestion buffer (0.01 M TFA, 0.2 M TRIS) were added to the cell pellets. Trypsinization was carried out by incubating the samples overnight at 37 °C. Subsequently, the pH values were reduced to 1–2 by adding 5 µL of TFA and the samples were spun down for 2 min at 3000 × *g* to only use the supernatant for further steps. The peptides were then desalted with the help of Pierce™ Peptide Desalting Spin Columns (Thermo Scientific) according to the manufacture’s protocol no. 2162704. The liquid contents of the desalted peptide eluates were evaporated by vacuum centrifugation (UNIVAPO 150 H, UniEquip) and the peptides were then dissolved in 20 µL 0.1% formic acid. Peptide concentrations were determined by absorbance measurements at 280 nm (NanoPhotometer NP80, Implen) and samples were diluted to a peptide concentration of 0.2 µg/µL using 0.1% formic acid.

### Liquid chromatography and mass spectrometry

Peptides were analyzed on an EASY-nanoLC 1200 (Thermo Fisher Scientific) coupled online to a Q Exactive™ HF mass spectrometer (Thermo Fisher Scientific). 1 µg of peptides were separated on a PepSep column (15 cm length, 75 μm i.d., 1.5 μm C18 beads, PepSep, Marslev, Denmark) using a stepped 30 min gradient of 80% acetonitrile (solvent B) in 0.1% formic acid (solvent A) at 300 nL/min flow rate: 4–9% B in 2:17 min, 9–26% B in 18:28 min, 26–31% B in 3:04 min, 31–38% B in 2:41 min, 39–95% B in 0:10 min, 95% B for 2:20 min, 95–0% B in 0:10 min and 0% B for 0:50 min. Column temperature was kept at 50 °C using a butterfly heater (Phoenix S&T, Chester, PA, USA). The Q Exactive™ HF was operated in a data-independent (DIA) manner in the m/z range of 345–1,650. Full scan spectra were recorded with a resolution of 120,000 using an automatic gain control (AGC) target value of 3 × 10^6^ with a maximum injection time of 100 ms. The full scans were followed by 39 DIA scans of dynamic window widths using an overlap of 0.5 Th^37^. DIA spectra were recorded at a resolution of 30,000 using an AGC target value of 3 × 10^6^ with the maximum injection time set to auto and a first fixed mass of 200 Th. Normalized collision energy (NCE) was set to 27% and default charge state was set to 3. Peptides were ionized using electrospray with a stainless-steel emitter, I.D. 30 μm (PepSep) at a spray voltage of 2.1 kV and a heated capillary temperature of 275 °C.

### Data analysis and visualization

Reference protein sequences of *Escherichia coli* (K12) (UP000000625) were obtained from UniProt^39^. Spectral libraries were predicted using the deep-learning algorithm implemented in DIA-NN (version 1.8.1)^40^ with strict trypsin specificity (KR not P) allowing up to one missed cleavage site in the m/z range of 350–1,150 with charges states of 2–4 for all peptides consisting of 7–30 amino acids with enabled N-terminal methionine excision and cysteine carbamidomethylation. The mass spectra were analyzed in DIA-NN (version 1.8.1) using default settings including a false discovery rate (FDR) of 1% for precursor identifications with enabled “match between run” (MBR) option for technical triplicates. The resulting pg_matrix.tsv (Lib.Q.Value = 1%) files were used for further analysis in Perseus (version 2.0.3.0)^41^.

In Perseus, the protein abundances were first transformed into log2 values. To address proteins which were not detected in each solute-adaptation experiment (from here on referred to as treatments), only proteins were kept which could at least be detected in all three triplicates of a single treatment. Missing values were then replaced using a normal distribution. Subsequently, the abundances were ANOVA tested (FDR ≤ 0.01) and an additional post hoc test (FDR ≤ 0.01) was performed in order to identify significantly different protein pairs for each treatment combination. After these analyses, only those proteins were kept for which at least one treatment exhibited a significantly different expression relative to the control. Further, the abundances of the biological triplicates were median averaged and log2-fold changes for each treatment relative to the control were calculated.

Next, we extracted sets of proteins involved in the adaptive response of *E. coli* to each of the three different treatments (NaClO_4_, NaCl and glycerol). To visualize this data, Venn diagrams were used, showing both up- and downregulated proteins exclusive to each solute, as well as those shared among multiple treatments (Fig. 1c). Proteins were assigned to the three treatments using a strict filtering process based on the information obtained by post-hoc testing. This statistical analysis yielded information on significantly differently expressed protein pairs between the three treatments and the control, as well as between the treatments. To capture the most distinctive adaptive responses and highlight the most significant differences between the treatments, two criteria had to be met: (1) The protein expression had to be significantly different from that of the control. (2) In such cases, the protein expression also had to be significantly different relative to the other treatments, in which it was not part of the adaptive response. As an example, proteins assigned to the adaptive response of exclusively NaClO_4_-induced stress were significantly up- or downregulated relative to the control as well as to NaCl and glycerol. At the same time in NaCl and glycerol, the protein expression was either not significantly different from that of the control, or, if it was, significantly downregulated when upregulated in NaClO_4_ or vice versa, ensuring that opposing regulations were not excluded in the treatment types.

The protein groups of interest were then used to identify significantly enriched pathways (FDR ≤ 0.05) using protein-protein interaction networks using the STRING database (version 11.5)^42^. This method allows protein clusters to be visualized and identifies significant functional enrichments among them. Protein clusters of interest were then annotated based on best encapsulating enrichments and high strength scores (log_10_ of observed proteins in network divided by expected number of proteins in a random network of the same size). A detailed summary of functional enrichments for each protein group network is listed in Supplementary Table S1-S8.

## Results

### Stepwise adaptation to elevated concentrations of NaClO_4_, NaCl and glycerol

To identify perchlorate-specific adaptation mechanisms of *E. coli*, we first used laboratory evolution to adapt the microbe to increasingly higher concentrations of NaClO_4_ (Supplementary Fig. S1a). With this approach, growth of *E. coli* at a concentration of 0.2 mol/kg NaClO_4_ was achieved (Fig. 1b). Notably, initial growth was followed by cell death and a secondary growth phase. Secondary growth phases were observed in several growth experiments conducted in perchlorate-containing media and might require further investigation and upcoming studies. A further attempt to increase the NaClO_4_ concentration to 0.225 mol/kg and 0.25 mol/kg resulted in culture growth, but was immediately followed by complete death of the culture with neither a stationary nor a secondary growth phase (Supplementary Fig. S1a). Thus, 0.2 mol/kg was chosen for proteome analysis.

Perchlorate induces various types of stress, including water activity, osmotic and ionic stress. We therefore additionally adapted *E. coli* to increasing NaCl and glycerol concentrations in separate approaches. This allowed us to compare the individual adaptive mechanisms, provided information on how *E. coli* reacts to different stresses, and enabled the extraction of those adaptive strategies that are likely to be perchlorate-specific. For NaCl, growth was detected up to a concentration of 1.4 mol/kg (Supplementary Fig. S1b). However, to achieve sufficient cell densities and more reproducible growth patterns for proteome analysis, the next lower concentration of 1.2 mol/kg NaCl was used. For glycerol, growth was observed up to a concentration of 4 mol/kg. Two further attempts to increase the concentration to 4.25 mol/kg resulted in substantial population decline, where one culture was able to recover slowly while the other died completely (Supplementary Fig. S1c).

To demonstrate that the adaptation approach contributed to better growth at the concentrations used for proteomics analysis, non-adapted *E. coli* was inoculated from optimal growth medium into the respective solute concentrations (Supplementary Fig. S1). It could be observed that each cell culture died within the first week and had cell densities below the detection limit. For *E. coli* in medium supplemented with 0.2 mol/kg NaClO_4_ and 4 mol/kg glycerol, no subsequent growth could be detected within the observed period of 17–20 days. While *E. coli* cultured in medium supplemented with 1.2 mol/kg NaCl initially died to a cell density below the detection limit, it was able to recover and a cell density of 10 CFU/mL could be measured after 17 days. Nevertheless, the stepwise adaptation approach led to enhanced growth and thus relevant adaptation-related phenotypic changes.

Accordingly, it is evident that *E. coli* could not grow at a concentration of 0.2 mol/kg NaClO_4_ without prior adaptation. In addition, we have demonstrated that a gradual increase in NaClO_4_ is necessary to achieve growth at this tolerance concentration. For this, we used a culture grown in 0.125 mol/kg NaClO_4_ as starting point and inoculated it into medium supplemented with 0.15 mol/kg and 0.175 mol/kg NaClO_4_ (Supplementary Fig. S1a). While growth was observed at 0.15 mol/kg, the culture in medium supplemented with 0.175 mol/kg NaClO_4_ died, highlighting the importance of gradual steps of concentration increase.

In all adaptation approaches, cell filamentation was observed, which was most pronounced in *E. coli* adapted to NaClO_4_ (Supplementary Fig. S2). Stress-induced regulation of cell proliferation, including replication arrest, sporulation and filamentation, is common in bacteria^43^. For example, cell filamentation has been reported in *E. coli* as a response to a variety of stressors, including UV-radiation^44^, antibiotics^45^ and chaotropic agents^46^. It has also been observed previously upon perchlorate-induced stress in *E. coli*^46^, as well as the desiccation tolerant bacterium *Hydrogenothermus marinus*^47^. These results prompt further investigation in the future but is not within the scope of this study, which focuses on the proteomic stress responses.

### Proteomic analysis of adaptive phenotypes

Cell cultures adapted to 0.2 mol/kg NaClO_4_, 1.2 mol/kg NaCl and 4 mol/kg glycerol, as well as the wild-type (WT) grown in solute-free medium and functioning as control, were inoculated in triplicates and harvested in the late exponential growth phase for proteome extraction and subsequent analysis.

**Fig. 2:**
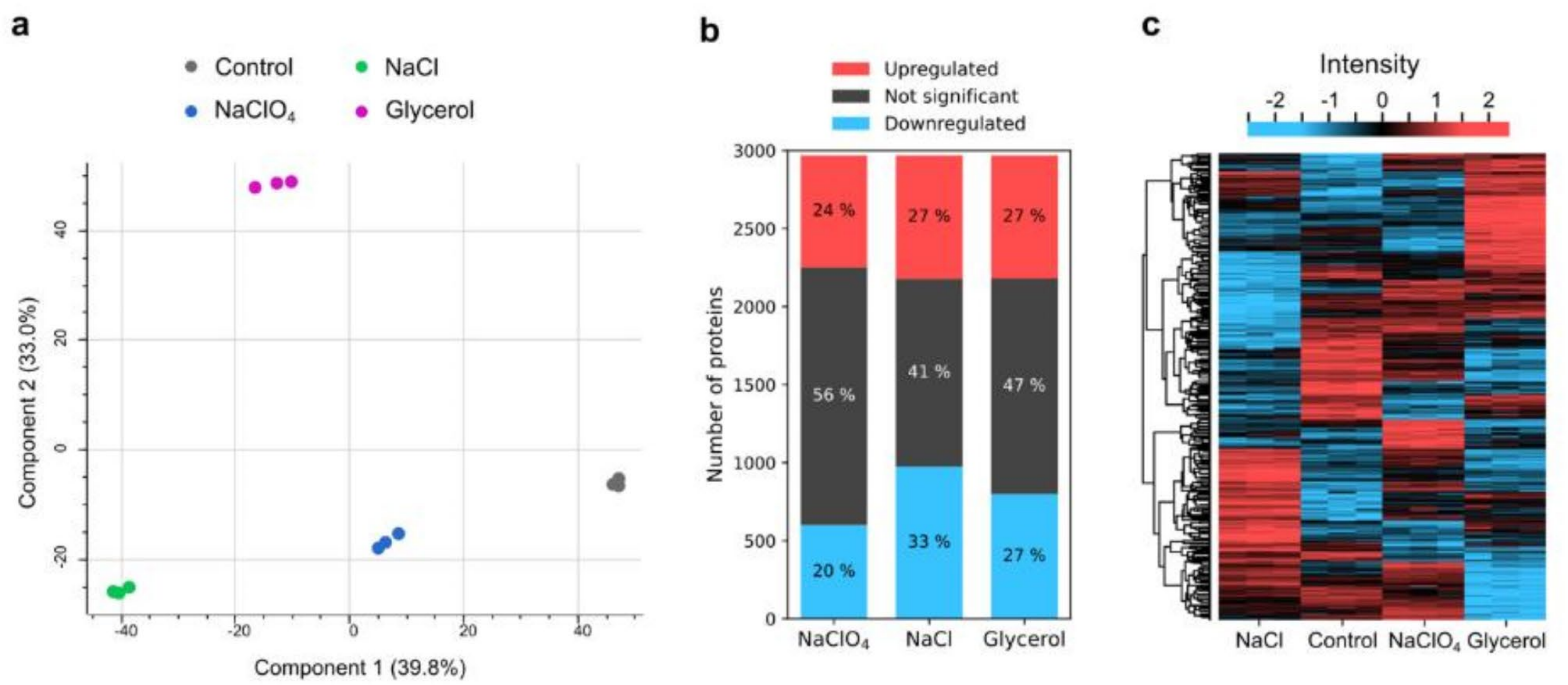
Overview of proteomic phenotypes after adaptation of *E. coli* to 0.2 mol/kg NaClO_4_, 1.2 mol/kg NaCl and 4.0 mol/kg glycerol. (**a**) Principal component analysis (PCA) depicting 72.8% of the total variance in the data. (**b**) Fractions of significantly regulated proteins of all proteins that were detected by LC-MS in each treatment. Significant regulation relative to the control was determined by ANOVA (FDR ≤ 0.01) and post hoc testing (FDR ≤ 0.01) (**c**) Hierarchical clustering of proteins passing both statistical tests. The protein abundances were log2-transformed and z-normalized, with downregulated proteins colored blue and upregulated ones colored red. The heatmap and PCA were generated by the Perseus software^41^.

Out of the 4437 proteins of *E. coli* K-12^39^, 2948 were detected in each triplicate of at least one treatment with the help of LC-MS, corresponding to a genome coverage of 66.4%. The protein abundances were log2-transformed and tested for statistical significance by an analysis of variance (ANOVA, FDR ≤ 0.01). This allowed the identification of a total of 2756 significantly differentially expressed proteins. A subsequent post hoc analysis (FDR ≤ 0.01) revealed a total of 2248 proteins to be significantly differently expressed between at least one treatment and the solute-free control. In summary, 24–27% of all detected proteins were significantly upregulated and 20–33% were significantly downregulated in the respective treatments (Fig. 2b). To better understand the structure and variance of the data, a principal component analysis (PCA) was performed. PC1 and PC2 scores were plotted, capturing 72.8% of the total variance in the data (Fig. 2a). PC1 can be used to capture the variance between all three treatments and the control, while PC2 is primarily useful to distinguish between glycerol and the other treatments. The reason for this may be that glycerol as a metabolite might interact with the cellular metabolism, thus causing substantially different proteomic changes compared to the treatments inducing ionic stress.

All significantly differently expressed proteins relative to the control were considered for further analyses. Protein expression patterns were plotted in a heatmap through hierarchical clustering (Fig. 2c). Clusters of shared adaptive responses of both up- and downregulated proteins between the solute-adaptation approaches were evident. However, it was also apparent that there were clear differences between the different solute-induced stress responses, with proteins that were more strongly regulated in one solute than the others or exhibited inverse expression patterns.

### Identification of perchlorate-specific adaptation mechanisms

To determine and filter those adaptive responses that were specific for perchlorates, the protein expression data was further processed. Post hoc testing (FDR ≤ 0.01) provided information on protein pairs that were significantly differently expressed between not only the three treatments and the control, but also between the treatments. This was used to extract sets of proteins which were significantly regulated as adaptative response to each solute relative to all the other ones, as well as those which were found to be significantly regulated under multiple treatments and thus belong to a shared stress response (Fig. 1c). With this method, 1819 proteins were grouped into the different protein sets, out of which 422 proteins were part of both a significantly up- as well as downregulated protein set of different treatments. Overall, 1110 proteins were assigned to upregulated sets of proteins and 1131 to downregulated ones.

**Fig. 3:**
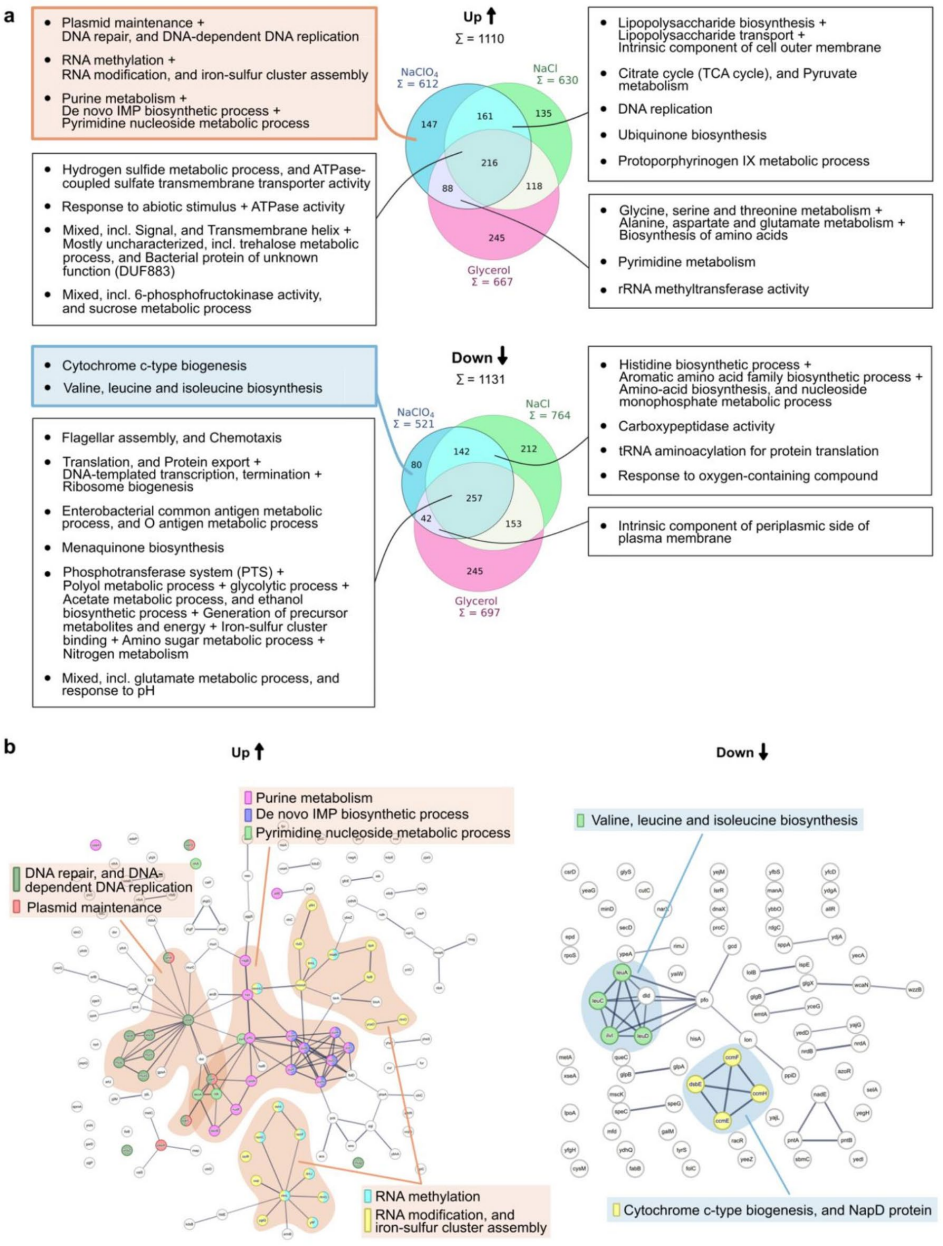
Proteomic adaptation mechanisms of *E. coli* to NaClO_4_ stress. (**a**) Sets of proteins that are part of solute-specific and shared proteomic adaptive responses were extracted using a strict filtering process (see Materials and Methods) and visualized using Venn diagrams for up- and downregulated proteins. Each protein set was then fed into the STRING database (Version 11.5)^42^, which was used to identify significant adaptive pathways (FDR ≤ 0.05) of protein clusters. The corresponding cluster annotations are listed for each protein set, with a plus indicating overlapping cluster annotations. For many clusters, several enrichments were found, which, however, were very general and can be found in Supplementary Table S2-S4. (**b**) Significantly enriched pathways of the NaClO_4_-specific upregulated (left) and downregulated (right) stress response as identified using the STRING database (Version 11.5)^42^. The protein clusters are outlined, and the significant pathways are annotated, with unique colors representing each enrichment.

Using this method, 612 significantly upregulated proteins relative to the control were detected for NaClO_4_ including those shared with NaCl and glycerol treatments (Fig. 3a). Out of these, 147 proteins were significantly upregulated exclusively in NaClO_4_, meaning that these were significantly upregulated relative to the control as well as relative to NaCl and glycerol. The number of downregulated proteins in NaClO_4_ was lower compared to the upregulated ones with 521 proteins in total, and 80 exclusively in the NaClO_4_ treatment. The number of proteins found in the parallel stress response to NaClO_4_ and NaCl is higher than to NaClO_4_ and glycerol with 135 compared to 88 shared upregulated proteins, respectively, and 212 compared to 42 shared downregulated proteins.

The proteins for specific and shared adaptive responses were used to identify functional pathway enrichments (FDR ≤ 0.05) using STRING^42^, a database containing predicted protein-protein associations and pathway annotations. With regard to the shared stress responses, it was apparent that between NaClO_4_ and NaCl it overlapped considerably more than between NaClO_4_ and glycerol. Figure 3b indicates the protein clusters and the associated pathway enrichments that were determined to be specific for NaClO_4_. The adaptive response focused on the upregulation of various pathways involving nucleic acids. DNA replication was found as shared adaptive response to NaClO_4_ and NaCl and pyrimidine metabolism as well as rRNA methylation as a shared response to NaClO_4_ and glycerol. However, exclusively in NaClO_4_, DNA repair, nucleotide biosynthesis pathways as well as non-coding RNA modifications were also upregulated, highlighting the role of nucleic acid repair and stability in perchlorate resistance. NaClO_4_-specifically downregulated pathways included cytochrome c-type biogenesis and branched-chain (valine, leucine and isoleucine) amino acids.

## Discussion

The objective of this study was to identify proteomic survival strategies of *E. coli* in response to elevated concentrations of perchlorate, in order to evaluate potential adaptation approaches of putative microorganisms in perchlorate-rich environments on Mars. Using a stepwise adaptation approach, *E. coli* was able to tolerate a NaClO_4_ concentration of up to 0.2 mol/kg (2.4 wt%) (Fig. 1b, Supplementary Fig. S1a). For comparison, the current record is held by the halotolerant yeast *D. hansenii* with growth at 2.5 mol/kg (23 wt%) NaClO_4_^33^. While *E. coli* is not the most perchlorate-tolerant species, its well-annotated genome and proteome made it ideal for the proteomic analyses central to this study. On Mars, highly concentrated brines may be formed under deliquescence^21,34^. For instance, a thermodynamic analysis determined that a perchlorate threshold concentration of 14.4 wt% NaClO_4_ enables liquid water to be stable under Martian atmospheric conditions in a narrow temperature range of approximately − 4.363 and − 4.336 °C, with the window widening for increasing concentrations^48^. While the perchlorate tolerance achieved in this study for *E. coli* would thus not support growth at concentrations in putative Martian brines, a stepwise adaptation approach exceeds tolerances without prior adaptation (Supplementary Fig. S1a). For *E. coli*, 0.125 mol/L NaClO_4_has been described previously as lethal perchlorate concentration^35^, while another study reported some growth before cell lysis at a concentration of 0.15 mol/L NaClO_4_^46^ (for comparison, at relatively low concentrations, the solution density is close to 1 kg/L, making mol/L ≈ mol/kg). Reducing incremental increase steps and increasing the number of generations may enable the adaptation to even higher concentrations. For example, How et al. were able to adapt *E. coli* to a NaCl concentration of 1.9 mol/L by increasing the concentration each month and subculturing three times per week, thereby exceeding its bacteriostatic threshold of 1.2–1.4 mol/L^49^. To be able to make statistical statements about the tolerances resulting from the incremental increase in solute concentrations, the adaptation experiment would have to be repeated several times. However, this was not the focus of the present study, which was instead centered on the proteomic stress response.

In addition to the NaClO_4_-exposed treatment, *E. coli* was subjected to increasing concentrations of NaCl and glycerol in two separate approaches. This laid the foundation for extracting those proteomic strategies that were unique to NaClO_4_-induced stress (Fig. 3). In all three adaptation approaches (NaClO_4_, NaCl, and glycerol), impairment of growth compared to the salt-free control was evident (Fig. 1b, Supplementary Fig. S1). Slower growth has been previously demonstrated to enhance adaptability^50^. Balancing growth and stress resistance is important for bacterial survival and is achieved through a shift in the allocation of resources^51^. The ribosomal protein fraction has been positively correlated with growth rate under changes in nutrient quality^52^. Here, we observed that translation and ribosome biogenesis was downregulated in all three treatments. Among others, the large subunit ribosomal proteins RplABCDEFIKLOVX and the small subunit ribosomal proteins RpsABDEHR were downregulated, as were the translation initiation factor InfC and elongation factor FusA. This may indicate that energy is redirected from growth-related pathways to those crucial for survival under the respective stress conditions.

In all three treatments, we found in the proteomic dataset an upregulation of environmental signal sensing and response. These include proteins involved in a response to osmotic stress (e.g. OsmE, OsmC and OtsA) and envelope stress (e.g. CpxAR), indicating a reaction to the different stressors in the environment. For instance, OtsA catalyzes the first step of trehalose biosynthesis, which is an osmoprotectant needed for turgor restoration at high osmolarity^53^. And CpxAR is a two-component system that senses envelope stress caused by misfolded periplasmic proteins and targets, among others, proteins involved in protein folding, proteolysis and membrane integrity^54^. Notably, the response to two separate environmental stresses was downregulated, indicating a potential adaptational trade-off caused by a reallocation of cellular resources. These were proteins associated with a response to pH stress across all treatments (e.g. acid stress protection proteins HdeAB), as well as to oxygen-containing compounds in samples containing NaClO_4_ or NaCl (e.g. formaldehyde detoxification proteins FrmAB, and peroxide detoxification proteins AhpCF and KatG).

In the following, the most important differently regulated biochemical pathways in response to NaClO_4_ are discussed with regard to their specificity and their overlap with those in response to NaCl and glycerol.

The downregulation of histidine biosynthesis has been observed in response to water activity stress caused by NaCl, and was similarly attributed to the reallocation of energy resources^55^. We could also observe the downregulation of histidine as well as aromatic amino acid biosynthesis in response to both NaCl and NaClO_4_ (HisBCDFGHI and AroADH). However, the regulation of amino acid biosynthesis in response to environmental perturbations is complex, as the biosynthesis of different amino acids may be upregulated while others are downregulated^55,56^. This could also be observed here, as a number of amino acid biosynthesis proteins were upregulated as a shared stress response to glycerol and NaClO_4_ (e.g. ArgG, AspC, DapEB, GlyA and TyrB). Interestingly, proteins associated with branched-chain amino acid biosynthesis (LeuACD and IlvI) were downregulated exclusively in response to NaClO_4_. This is in contrast to an observation by Horinouchi et al., who found increased biosynthesis of histidine, tryptophan, and branched-chain amino acids in *E. coli* adapted to the chaotropic agent ethanol^57^. They also found that supplementing the medium with these amino acids causes enhanced ethanol-tolerance. This highlights the complexity of amino acid biosynthesis regulation in response to stress.

As a shared stress response to NaClO_4_ and NaCl, we found significant pathway enrichments that indicate an upregulation of aerobic respiration and downregulation of anaerobic pathways. For instance, in all three treatments, proteins involved in glycolysis (FbaA, GpmM, GapA, Pgk, TpiA) as well as acetate and ethanol production (PflB, Pta, Acka and AdhE) were downregulated. The pyruvate formate-lyase PflB anaerobically converts pyruvate and coenzyme-A to formate and acetyl-CoA. The latter can then be used to yield fermentation products through reduction to ethanol by AdhE or acetate by Pta and AckA^58^. In contrast, in response to elevated NaClO_4_ and NaCl, the pyruvate dehydrogenase complex (AceEF and Lpd) and metabolic downstream proteins of the tricarboxylic acid cycle were upregulated (AcnAB, SdhABC, MaeAB and SucABD). These significant enrichments thus demonstrate a change in the metabolic conversion of pyruvate towards aerobic respiration. In addition, a reduction in the expression of proteins involved in the biosynthesis of menaquinone (MenBCDE), which is linked to fermentation and anaerobic respiration, was observed^59^. In contrast, ubiquinone is associated with aerobic respiration and its biosynthesis proteins were upregulated (UbiBEFJ). Protoporphyrinogen IX metabolic process was also found to be upregulated in response to NaClO_4_ and NaCl (HemEGHXY), which is a precursor of heme and thus generally needed for respiration^60^. Furthermore, exclusively in NaClO_4_, we could observe the downregulation of proteins involved in cytochrome c-type biogenesis (CcmEFH and DsbE). C-type cytochromes are associated with respiration and induced under anaerobic conditions^61^, which further highlights the downregulation of anaerobic energy metabolism. This observation may seem counterintuitive, as aerobic respiration was shown to be downregulated in response to multiple stressors in *E. coli*^56^. Respiration is associated with a higher ATP-yield than fermentation. However, fermentation is more proteome efficient, which is why *E. coli* switches to fermentative pathways when the proteomic demand changes at higher growth rates to account for increased biomass synthesis^62^. Interestingly, in preliminary experiments (data not shown here) with non-adapted *E. coli* cells, we were able to observe NaClO_4_-specific upregulated fermentation processes alongside a downregulation of proteins related to aerobic metabolism. These results may indicate that *E. coli* favors aerobic respiration only after long-term adaptation, while non-adapted *E. coli* cells shocked with NaClO_4_ favor anaerobic respiration.

As the most pronounced NaClO_4_-specific stress response, we identified several upregulated enrichments involving nucleic acid repair and stabilization, indicating that nucleic acids were particularly affected by NaClO_4_. The reason for this may be enhanced chaotropic and/or oxidative stress, although the role of perchlorate-induced oxidative stress is still unclear^26^. It has been established that osmotic stress generally induces cross-protection against oxidative stress^63^, and we do find evidence of this in our data. In all treatments, hydrogen sulfide metabolism (CysDHIJNP) was upregulated, which is important for oxidative stress protection^64^. Additionally, DNA repair proteins were upregulated in all samples, such as recombination and repair proteins RecACN and excinuclease system proteins UvrAB. Furthermore, as a shared stress response to NaClO_4_ and NaCl, stress-induced DNA replication and repair proteins were upregulated, such as DNA polymerase II (PolB). In addition, pyrimidine metabolism (CarAB, MazG, Ndk, ThyA and UmpG) and rRNA methyltransferase activity (RlmEFC and RsmHG) were upregulated as a shared stress response to NaClO_4_ and glycerol. The upregulation of nucleotide biosynthesis may indicate an increased demand for nucleotides due to nucleic acid damage. RNA methylation plays an important role in response to various environmental stresses in *E. coli* due to its stabilizing effect^65^.

Exclusively in response to NaClO_4_, however, we found additional significantly upregulated enrichments with regard to nucleic acids. First, several DNA repair and replication proteins were upregulated, including DNA mismatch repair proteins MutLSY, DNA polymerase and exonuclease PolA and DNA topoisomerase IV complex (ParCE). Second, we found the upregulation of further RNA methylation proteins (MiaB, MnMG, TrmL, RlmGJL, RsmCFI and YfiF). And third, nucleotide biosynthesis pathways were significantly upregulated. Within these enrichments, de novo inosine monophosphate (IMP) biosynthesis (PurBDFHKNT), a purine precursor, is particularly noteworthy. Our results are similar to a study by Díaz-Rullo et al., who applied a metagenomics approach to identify perchlorate-resistance mechanisms in microbes from sediments of a hypersaline pond in the Atacama Desert. Similarly, they identified several genes involved in processes such as DNA protection, tRNA modification as well as a gene putatively coding for IMP dehydrogenase^35^.

In contrast, metabolic enrichments indicating nucleotide acid damage exclusively in response to NaCl or glycerol were less clear (Supplementary Fig. S3). In the case of glycerol, pathways that may be related to oxidative stress were found to be upregulated, including iron-sulfur cluster assembly (ErpA, GrxAD, IscAUX and NfuA) and the pentose phosphate pathway (e.g. Gnd, TalB and Zwf). An increase in flux through the pentose phosphate pathway may assist in an oxidative stress response by increasing the NADPH supply^66^. Furthermore, iron-sulfur clusters can be damaged by oxidants, which is why repair and de novo mechanisms play a role in oxidative stress^67^. This is further evidenced by the upregulation of multiple proteins involved in peroxide detoxification (e.g. AhpCF, BtuE and KatG). However, it is noteworthy that several pathways were also found to be downregulated in response to glycerol as well as NaCl. Purine biosynthesis and salvage (e.g. GuaD and XdhBCD) were significantly downregulated in response to glycerol and pyrimidine biosynthesis (e.g. CarAB and PyrBCH) in response to NaCl. In addition, the downregulation of the DNA restriction-modification system (HdsMRS and McrAB) as well as RNA processing and methylation (e.g. RlmDGIJL, RluDE and RsmAF) was a shared response to both NaCl and glycerol. Thus, it is clear that NaClO_4_ presumably exerts more oxidative and/or chaotropic stress on *E. coli* than NaCl and glycerol. Hence, to further understand these findings, it is of great interest in future experiments to compare the stress response of *E. coli* to perchlorates with that to other oxidizing and chaotropic agents.

In a previous study, the proteomic adaptation strategies of the halotolerant yeast *D. hansenii* exposed to NaClO_4_were identified^26^. Contrary to what we observed here, the stress response of the yeast focused on protein glycosylation and cell wall remodulation to stabilize biomacromolecules, presumably to counteract NaClO_4_-induced chaotropic stress. Though we were able to find significant enrichments indicating remodulation of the cell wall and membrane, these were not exclusive or more pronounced in response to NaClO_4_. For instance, in all treatments, enterobacterial common antigen and O antigen metabolic processes (Glf, RfbABCD, Rffg, WbbIK and WecBCDF) as well as chemotaxis and flagellar assembly (e.g. CheAWY, FlgDEFIK, FlhAC and FliACDGHIKLMNO) were downregulated. The downregulation of flagellar assembly has been observed to various stressors in *E. coli* and may be attributed to energy reallocation^56^. Furthermore, in response to both NaClO_4_ and NaCl, additional indications for cell envelope remodulations were observed, such as the downregulation of carboxypeptidase activity (AmpH, LdcA, LdtBD, MpaA and PbPG) and the upregulation of lipopolysaccharide biosynthesis (LptBCDFG and MsbA) and transport (LbxBK, KdsB and WaaQUYZ).

Perhaps, *E. coli* is not able to build up protection comparable to the cell wall and protein modifications of *D. hansenii*, which is why alleviating DNA and RNA structure damage is the predominant adaptive strategy of *E. coli* in response to NaClO_4_-induced stress. This indicates that different organisms, as in this specific case of different domains of life, exhibit distinct perchlorate-specific adaptation mechanisms. In addition, it has been hypothesized that perchlorate may affect the respiratory chain through oxidative interaction with redox-sensitive proteins, affecting predominantly mitochondria in eukaryotes but the whole cell envelope in prokaryotes, thereby reducing perchlorate tolerance to a greater extent in prokaryotes^34^. This suggests that different organisms likely have very different approaches and adaptive priorities in response to elevated perchlorate concentrations. Further studies in this area are necessary to gain a more comprehensive understanding. Besides using different model organisms, it is also of relevance to study the microbial tolerance and adaptation to other hygroscopic salts on Mars, such as chlorates, as well as to simulated Mars-like habitats, such as the Martian shallow subsurface^68^.

## Conclusion

This is the first study dealing with proteomic adaptation mechanisms important for a non-halotolerant microbe to survive under perchlorate-stress. We were able to adapt *E. coli* to grow at 0.2 mol/kg NaClO_4_, a concentration which would not have been tolerated without stepwise adaptation. Looking at this on a larger scale, putative Martian organisms, which may have evolved billions of years ago, would have had a much longer time span of at least several millions of years to adapt to the changing environmental conditions and desiccation of the planet, and thus to perchlorate-brines as one of the last potential habitats^16^. The adaptive strategy of *E. coli* focused on DNA repair and RNA stabilization. This is presumably to alleviate the enhanced chaotropic and/or oxidative stress perchlorate exerted on nucleic acids. This lays the foundation for future experiments, which should aim to further identify the role of oxidative and chaotropic stress of perchlorates. This adaptive strategy is therefore important for putative microorganisms living in near-surface perchlorate-rich brines on Mars as well as for the synthetic generation of perchlorate-resistant strains, which could play a role for bioremediation processes and ISRU of future human missions to Mars^17,18^. Future experiments may also include genomic analyses to evaluate adaptations occurring on the genome level during long-term adaptation and repeated adaptation experiments to statistically identify perchlorate tolerances.

## Electronic supplementary material

Below is the link to the electronic supplementary material.

Supplementary Material 1

Supplementary Material 2

## Data Availability

The mass spectrometry proteomics data have been deposited to the ProteomeXchange Consortium via the PRIDE^69^ partner repository with the dataset identifier PXD055273 and can be accessed at: https://www.ebi.ac.uk/pride/archive/projects/PXD055273. All other data is provided within the manuscript or supplementary information files.
