# Supplementary material for: Proteomic insights into survival strategies of Escherichia coli in perchlorate-rich Martian brines
Source: Sci Rep. 2025 Feb 27;15:6988. doi: 10.1038/s41598-025-91562-3 (PMC11865474; doi:10.1038/s41598-025-91562-3)
Supplement: Supplementary file 1 — Supplementary Material 1 [file 41598_2025_91562_MOESM1_ESM.docx]

**Supplementary Information**

Proteomic Insights into Survival Strategies of *Escherichia coli* in Perchlorate-Rich Martian Brines

Lea D. F. Kloss^1,#^, Joerg Doellinger^2^, Anne Gries^1^, Elisa Soler^1^, Peter Lasch^2^, Jacob Heinz^1*^

^1^Center for Astronomy and Astrophysics, RG Astrobiology, Technische Universität Berlin, Berlin, Germany

^2^Robert Koch-Institute, Centre for Biological Threats and Special Pathogens, Proteomics and Spectroscopy (ZBS6), Berlin, Germany

^#^Current affiliation: Institute for Computer Science & Department of Biology, Heinrich Heine University, Düsseldorf, Germany

^*^Correspondence: [heinz@tu-berlin.de](mailto:heinz@tu-berlin.de) or [jacobheinz@web.de](mailto:jacobheinz@web.de)


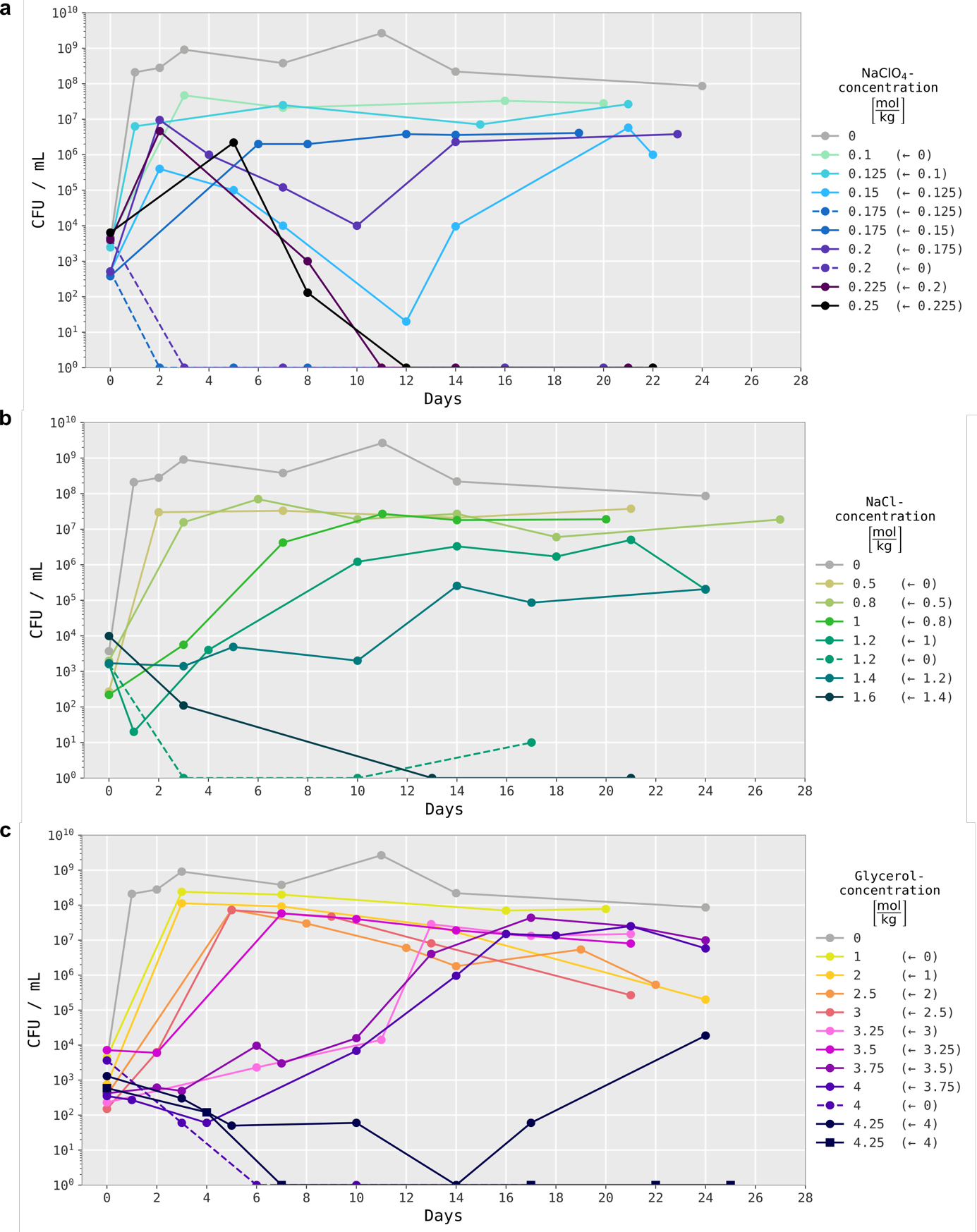


**Figure S1: Growth curves obtained during stepwise adaptation of *E. coli* to elevated concentrations of (a) NaClO_4_, (b) NaCl and (c) glycerol.** Each curve shows an example growth profile of a single cell culture at the respective solute and concentration. For each solute, the curves were generated from separate experimental runs, rather than from a single continuous experiment. The legend contains the information from which concentration each sample was inoculated in brackets. If no colony grew for undiluted plating, the cell density was below the detection limit and was set to 1 CFU/mL to show a cell density of 10^0^ CFU/mL in the logarithmic scale of the plot.


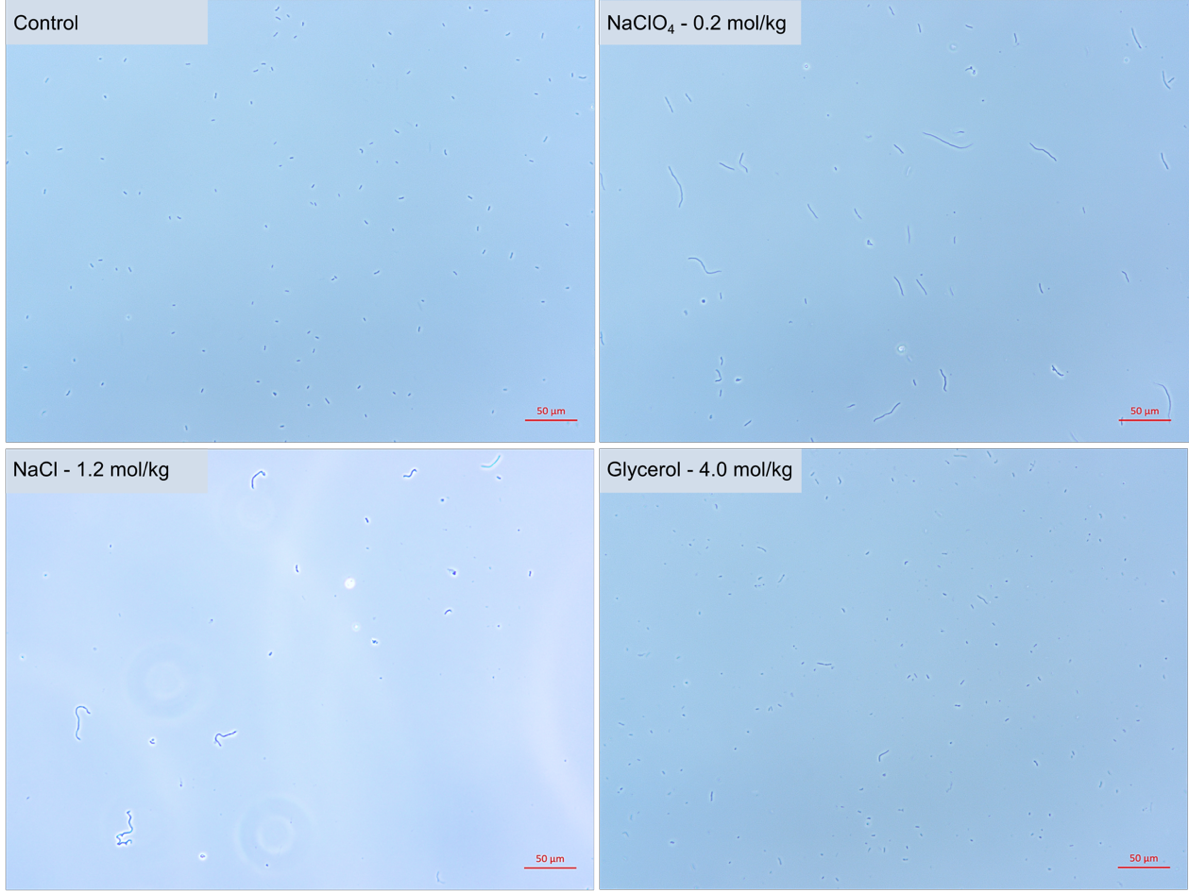


**Figure S2: Morphology of WT *E. coli* and after its stepwise adaptation to elevated concentrations of NaClO_4_, NaCl and glycerol.** Representative light microscopy images were taken during the late exponential growth phase.


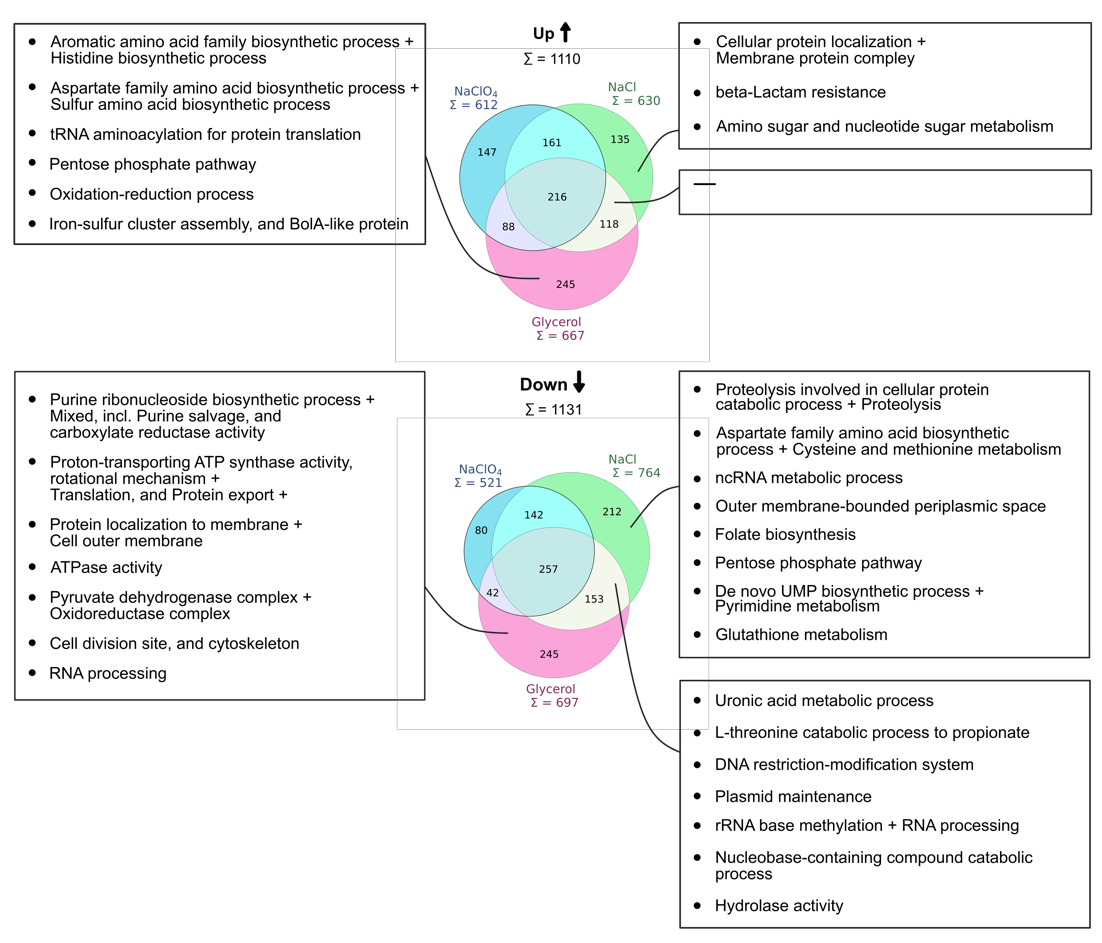


**Figure S3: Proteomic adaptation mechanisms of *E. coli* not part of the response to NaClO_4_.** Sets of proteins that are part of solute-specific and shared proteomic adaptive responses were extracted using a strict filtering process (see Materials and Methods) and visualized using Venn diagrams for up- and downregulated proteins. Each protein set was then fed into the STRING database (Version 11.5) (Szklarczyk et al., 2021), which was used to identify significant adaptive pathways (FDR ≤ 0.05) of protein clusters. The corresponding cluster annotations are listed for each protein set, with a plus indicating overlapping cluster annotations. For many clusters, several enrichments were found, which, however, were very general and can be found in Supplementary Tables S2-S4.
